# Geospatial analysis of surface water and groundwater quality using GIS in Ishaka subcounty, Bushenyi District, Uganda

**DOI:** 10.1038/s41598-026-43003-y

**Published:** 2026-03-02

**Authors:** Abdulkadir Ahmed Mohamed, Ambiga Kannapiran, Ahmed Mohamed Suliman Badawi, Supriya S

**Affiliations:** 1 Water Resources Engineering, Department of Civil Engineering, Kampala International University, Western Campus, Ishaka, Uganda; 2 Department of Civil Engineering, Kampala International University, Western Campus, Ishaka, Uganda; 3 Department of Chemistry, Jansons Institute of Technology, Coimbatore, Tamilnadu India

**Keywords:** Water quality, Physicochemical parameters, Biological parameters, Sample Sites, GIS mapping, WHO standards, Environmental sciences, Natural hazards

## Abstract

Access to safe drinking water remains a major public health challenge in low- and middle-income countries, particularly in rapidly urbanizing peri-urban settings. This study assessed the spatial variability of physicochemical and microbiological water quality in Ishaka Subcounty, western Uganda, using field-based measurements integrated with Geographic Information Systems (GIS). A total of 49 water samples were analysed for physicochemical parameters (pH, electrical conductivity, total dissolved solids, total suspended solids, temperature, dissolved oxygen, and nitrates) and microbiological indicators (faecal coliforms and *Escherichia coli*). Spatial interpolation was performed using the Thin Plate Spline method. Results indicate that physicochemical parameters largely complied with WHO and Uganda National Bureau of Standards drinking-water guidelines, while microbiological contamination was widespread and spatially clustered. These findings demonstrate that microbial contamination poses the most significant public health risk despite generally acceptable chemical water quality. The study provides spatially explicit evidence to support targeted water safety interventions and public health planning in similar peri-urban environments.

## Introduction

Access to safe drinking water remains a fundamental determinant of public health, socioeconomic development, and environmental sustainability worldwide. Although global efforts have substantially increased access to improved water sources over the past decades, water quality deterioration continues to pose serious risks, particularly in low- and middle-income countries^1^. The World Health Organization (WHO) estimates that unsafe drinking water contributes to hundreds of thousands of preventable deaths annually, primarily through waterborne diseases such as diarrhoea, cholera, typhoid, and dysentery^2^. These health impacts are most severe among vulnerable populations, especially children under five years of age^3^.

Recent studies consistently demonstrate that groundwater and surface water quality in rapidly urbanising regions is shaped by complex interactions between hydrogeochemical processes, anthropogenic activities, and inadequate sanitation infrastructure^4^. Spatial analyses using GIS-based interpolation techniques reveal significant heterogeneity in contaminant distribution, with physicochemical parameters such as pH, electrical conductivity, total dissolved solids, and major ions often masking localized zones of elevated health risk^5^. Furthermore, integrated assessments combining chemical indicators with microbial contamination and human health risk modelling show that reliance on compliance-based reporting alone may underestimate non-carcinogenic and carcinogenic risks, particularly in peri-urban and high-density setting^6^. These findings underscore the importance of spatially explicit, multi-indicator water quality evaluation frameworks to identify contamination hotspots and support risk-informed water resource management and public health protection^6^.

Beyond direct health impacts, deteriorating drinking water quality places substantial economic and social burdens on affected communities by increasing healthcare costs, reducing productivity, and constraining educational outcomes^7^. Recent global assessments indicate that the burden of unsafe water is closely linked to inadequate sanitation, poor wastewater management, and weak water quality monitoring frameworks, particularly in rapidly urbanising and peri-urban regions of Sub-Saharan Africa^8^. Even where access to “improved” water sources has expanded, microbial contamination and emerging chemical pollutants continue to undermine water safety, highlighting a critical gap between infrastructure provision and effective water quality management^9^.

In Sub-Saharan Africa, the challenge of safe drinking water is compounded by rapid population growth, unplanned urban expansion, limited sanitation infrastructure, and weak regulatory enforcement^10^. Many communities rely on shallow groundwater systems and surface water sources that are highly vulnerable to contamination from pit latrines, agricultural runoff, informal waste disposal, and livestock activities^11^ While groundwater is often perceived as naturally protected, increasing evidence demonstrates that microbial and chemical contaminants can readily migrate through subsurface pathways, particularly in areas with shallow water tables, fractured aquifers, and high population density^12^.

Recent research has highlighted the significance of microbial and viral contamination pathways in groundwater systems^13^. Pathogens can infiltrate aquifers through leaching from latrines, seepage from wastewater infrastructure, and preferential flow through fractures and macropores, undermining the assumed safety of groundwater supplies^14^. These findings underscore the need for integrated water quality assessments that move beyond chemical parameters alone to include microbiological indicators capable of capturing direct public health risks^15^.

Uganda exemplifies many of these regional water quality challenges. Despite notable progress in expanding access to improved water sources through national policies and infrastructure investments, numerous studies report persistent non-compliance with WHO and Uganda National Bureau of Standards (UNBS) drinking-water guidelines^16^. Elevated levels of turbidity, nutrients, dissolved solids, and faecal indicator bacteria have been documented in both surface water and groundwater sources across urban and peri-urban settings^17^. Rapid urbanisation, peri-urban expansion, and population pressure have increased wastewater generation and surface runoff, placing additional stress on aging water supply and sanitation systems^18^.

Physicochemical parameters such as pH, electrical conductivity, total dissolved solids, temperature, dissolved oxygen, and nutrients provide essential information on hydrogeochemical processes, mineralisation, and overall water chemistry. However, compliance with physicochemical standards alone does not guarantee water safety^19^. Microbiological indicators, particularly faecal coliforms and *Escherichia coli*, are widely recognised as direct measures of faecal contamination and public health risk^20^. Studies across Sub-Saharan Africa consistently report that microbial contamination often persists even when physicochemical parameters remain within acceptable limits, highlighting the importance of integrated assessment approaches^21^.

Ishaka Subcounty, located in Bushenyi District in western Uganda, represents a typical mixed urban–peri-urban environment where water quality challenges converge^22^. The subcounty relies on multiple water sources, including piped water supplied by the National Water and Sewerage Corporation (NWSC), boreholes, protected and unprotected springs, shallow wells, and rainwater harvesting systems^23^. Although the NWSC network has improved physical access to water, supply interruptions and quality concerns frequently compel households to depend on alternative sources of uncertain safety^24^. Previous studies in Bushenyi District have reported widespread bacteriological contamination linked to inadequate sanitation facilities, unlined pit latrines, and surface runoff during rainfall events^25^.

Understanding the spatial variability of water quality across different source types and geographic locations is critical for effective water resource management and public health protection^26^. Traditional water quality assessments based on isolated sampling points provide limited insight into spatial patterns, contamination gradients, and high-risk zones^27^. Geographic Information Systems (GIS) offer a powerful framework for integrating field-based water quality data with spatial analysis, enabling the visualisation of contamination hotspots and supporting evidence-based decision-making^28^.

GIS-based interpolation techniques have been increasingly applied in surface water and groundwater quality studies to estimate spatial distributions of water quality parameters, particularly in data-scarce regions. Methods such as inverse distance weighting, kriging, and spline interpolation have demonstrated utility in identifying contamination trends and guiding targeted interventions^29^. When combined with physicochemical and microbiological indicators, GIS analysis enhances understanding of contamination dynamics and their potential links to land use, sanitation practices, and hydrogeological conditions^30^.

Recent advances in water quality modelling emphasise the integration of multiple indicators to capture complex contamination processes. Studies have demonstrated that parameters such as pH, electrical conductivity, total dissolved solids, suspended solids, nutrients, and faecal indicator bacteria collectively provide a robust representation of water quality status and public health risk^31^. Spatial analysis of these parameters allows for identification of zones where chemical stability masks underlying microbial hazards, a phenomenon commonly observed in peri-urban groundwater systems^32^.

In such settings, reliance on physicochemical compliance alone may lead to an underestimation of health risks, as microbiological contamination often exhibits high spatial heterogeneity driven by sanitation infrastructure, land use practices, and subsurface transport pathways^33^. GIS-based approaches enable the integration of point-based measurements into continuous spatial surfaces, improving the detection of contamination hotspots and supporting risk-informed water management decisions^34^. This integrated framework is particularly valuable in data-limited regions, where targeted interventions depend on understanding both the spatial distribution and interaction of chemical and microbial water quality indicators^35^.

Despite the growing body of research on GIS-based water quality assessment, limited studies have systematically evaluated both surface water and groundwater quality in Ishaka Subcounty using integrated physicochemical, microbiological, and spatial analysis approaches. Existing studies often focus on individual parameters or single water source types, providing fragmented evidence that is insufficient for comprehensive water safety planning^36^. Furthermore, few studies in the region explicitly examine spatial contamination patterns in relation to public health risk using GIS-based interpolation techniques^37^.

In addition, the majority of available assessments rely on point-based compliance reporting, which does not adequately capture spatial heterogeneity or localized contamination dynamics. Such approaches may overlook micro-scale hotspots where water quality deterioration poses disproportionate health risks, particularly in peri-urban environments characterized by heterogeneous land use, variable sanitation coverage, and complex hydrogeological conditions^38^. The absence of spatially integrated analyses limits the ability of policymakers and water managers to priorities interventions based on risk severity and geographic vulnerability^39^.

There is therefore a critical need for a spatially explicit assessment that compares multiple water sources against national and international drinking-water standards while identifying contamination hotspots and potential risk drivers^40^. Such an assessment can provide valuable evidence to inform water safety planning, sanitation improvement, and public health interventions in Ishaka Subcounty and similar peri-urban settings across Sub-Saharan Africa^41^.

### Objectives of the Study

The main objective of this study was to assess the spatial variability of surface water and groundwater quality in Ishaka Subcounty using physicochemical, microbiological, and GIS-based analysis.

The specific objectives were to:

- assess the physicochemical quality of selected water sources relative to WHO and UNBS drinking-water standards;
- evaluate microbiological water quality using faecal indicator bacteria;
- analyse the spatial distribution of key water quality parameters using GIS-based interpolation techniques; and.
- identify contamination hotspots and implications for water resource management and public health.

## Materials and methods

### Study Area

**Fig. 1:**
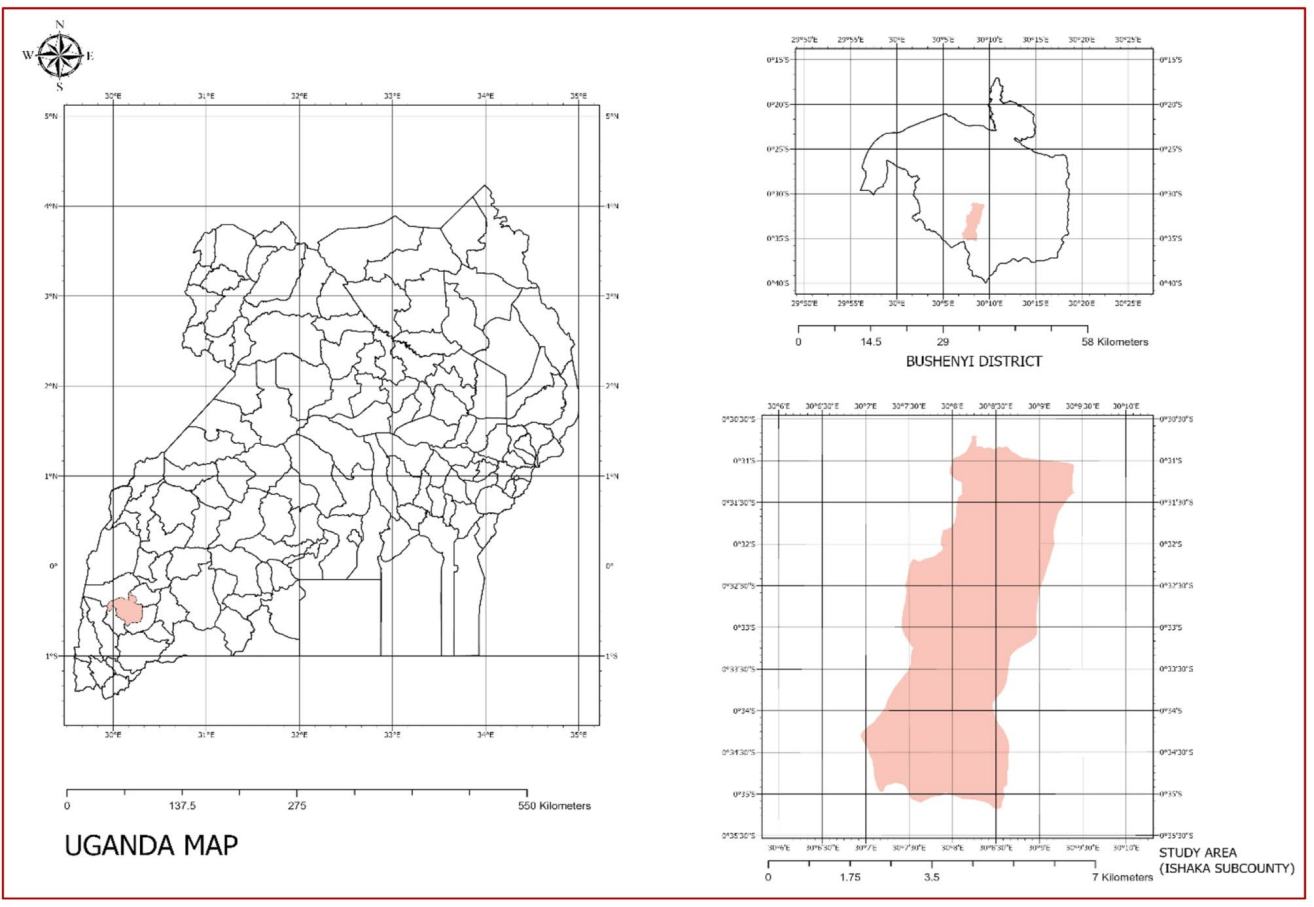
Study Area.

The study was conducted in Ishaka Subcounty, located in Bushenyi District in south-western Uganda, approximately 300 km southwest of Kampala^42^. The area exhibited a mixed urban–peri-urban setting characterised by rapid population growth, variable sanitation coverage, and dependence on multiple drinking-water sources^43^. The climate was tropical, with bimodal rainfall patterns and moderate temperatures throughout the year^44^. Groundwater was mainly accessed through shallow wells, boreholes, and springs, while piped water was supplied by the National Water and Sewerage Corporation (NWSC)^45^. Sanitation infrastructure was dominated by pit latrines, which have been reported to pose contamination risks to shallow groundwater systems in densely populated areas^46^. The location of the study area and sampling points is shown in Fig. 1.

### Study Design

A cross-sectional study design was adopted to assess the spatial variability of drinking-water quality across Ishaka Subcounty^47^. This design was selected to capture spatial differences in physicochemical and microbiological parameters at a single point in time rather than temporal variation across seasons^48^. Cross-sectional approaches are widely used in baseline water quality assessments, particularly in data-limited settings^49^.

### Sampling Design and Site Selection

A total of forty-nine (49) water samples were collected across Ishaka Subcounty to capture spatial variability in both surface water and groundwater quality. The sample size was determined based on field accessibility, logistical feasibility, and the relative reliance of local communities on different water sources^50^.

Sampling sites were distributed to ensure spatial coverage across densely populated, peri-urban, and peripheral zones of the subcounty^51^. Greater emphasis was placed on widely used sources to reflect population exposure rather than proportional representation by source type alone^52^. This approach ensured that areas with higher public health relevance were adequately represented while maintaining spatial balance across the study area^53^.

### Sampling Frequency

Water sampling was conducted as a cross-sectional survey during a single sampling campaign^54^. The objective was to assess spatial differences in water quality rather than temporal variability^55^. Although seasonal variation was not explicitly assessed, the sampling period was selected to represent typical water use conditions. The results therefore provide a snapshot of water quality conditions at the time of sampling^56^.

### Sample Collection

Water samples were collected following World Health Organization (WHO) guidelines for drinking-water quality assessment^57^. Sterile polyethylene bottles were used for physicochemical analyses, while sterilised glass bottles were used for microbiological sampling. Prior to sample collection, taps were allowed to run for several minutes to flush stagnant water. Samples from wells and springs were collected carefully to avoid sediment disturbance^58^.

All sampling points were georeferenced using a handheld Global Positioning System (GPS) device. Samples were stored in ice-cooled containers and transported to the laboratory for analysis within six hours of collection to minimise physicochemical and microbiological changes^59^.

### Physicochemical Analysis

Physicochemical parameters analysed included pH, electrical conductivity (EC), total dissolved solids (TDS), total suspended solids (TSS), dissolved oxygen (DO), and nitrates. pH was measured using a calibrated digital pH meter, electrical conductivity using a portable conductivity meter, and dissolved oxygen using a digital DO meter^60^. Total dissolved solids and total suspended solids were determined using standard gravimetric methods, while nitrate concentrations were analysed using spectrophotometric techniques^61^.

All analyses followed standard methods recommended by the WHO and the Uganda National Bureau of Standards (UNBS) for drinking-water quality assessment^62^.

### Microbiological Analysis

Microbiological water quality was assessed by analysing faecal coliforms and *Escherichia coli* using the membrane filtration technique. Water samples were filtered through sterile 0.45 μm membrane filters and incubated on selective media under controlled laboratory conditions^63^. Colony-forming units (CFU) were counted and expressed as CFU per 100 mL of water. Results were evaluated against WHO and UNBS drinking-water standards, where the presence of *E. coli* indicates faecal contamination and unacceptable water quality^64^.

### Quality Assurance and Quality Control (QA/QC)

To ensure data reliability and reproducibility, rigorous quality assurance and quality control (QA/QC) procedures were implemented throughout sampling and laboratory analysis. All instruments were calibrated daily prior to use following manufacturer specifications^65^. Duplicate samples were analysed to assess analytical precision, and blank samples were included to detect potential contamination during sample handling and analysis. Calibration checks were maintained within ± 5% of reference standards. Laboratory procedures followed standardised protocols to ensure consistency and comparability of results^66^.

### Spatial Analysis

Geospatial analysis was performed using ArcGIS Pro. Spatial interpolation of water quality parameters was conducted using the Thin Plate Spline (TPS) method. TPS was selected because of its ability to generate smooth surfaces from irregularly distributed sampling points and its suitability for environmental variables that exhibit gradual spatial transitions^67^. Compared to kriging or inverse distance weighting (IDW), TPS requires fewer assumptions regarding spatial stationarity and performs well when sample density is moderate^68^. While formal cross-validation metrics such as RMSE were not applied, the method provided consistent and interpretable spatial patterns suitable for exploratory contamination hotspot identification^69^.

Compared to other methods, thin plate splines (TPS) prioritize global smoothness over local accuracy. Unlike Inverse Distance Weighting (IDW), which can create localized “bullseye” patterns, TPS bends a continuous surface through all points, minimizing abrupt changes^67^. However, unlike Kriging, TPS is a deterministic method that provides no uncertainty estimates and is more sensitive to outliers, as its global calculation can cause erratic extrapolation in data-sparse areas.

The spatial interpolation results should therefore be interpreted as exploratory rather than predictive, given the absence of formal cross-validation metrics such as RMSE.

### Statistical Analysis

Descriptive statistical analysis was performed to summarise water quality parameters, including minimum, maximum, mean, and standard deviation values. Correlation analysis was conducted to explore relationships between selected parameters, including electrical conductivity and total dissolved solids, pH and electrical conductivity, and total suspended solids and microbial indicators^70^. Statistical analyses were performed using standard spreadsheet and statistical software packages, following established approaches in environmental water quality studies^71^.

### Ethical Considerations

The study did not involve human subjects or personal data. Permission for water sampling was obtained from relevant local authorities and community leaders prior to data collection. All sampling activities were conducted in accordance with local regulations and ethical research practices.

## Results

### pH

Most pH measurements complied with the WHO and UNBS recommended range of 6.5–8.5, while a smaller proportion fell below the lower guideline limit (Table 1). Overall pH values ranged from 5.18 to 7.79, indicating heterogeneity in water chemistry across the study area rather than uniform conditions.

**Fig. 2:**
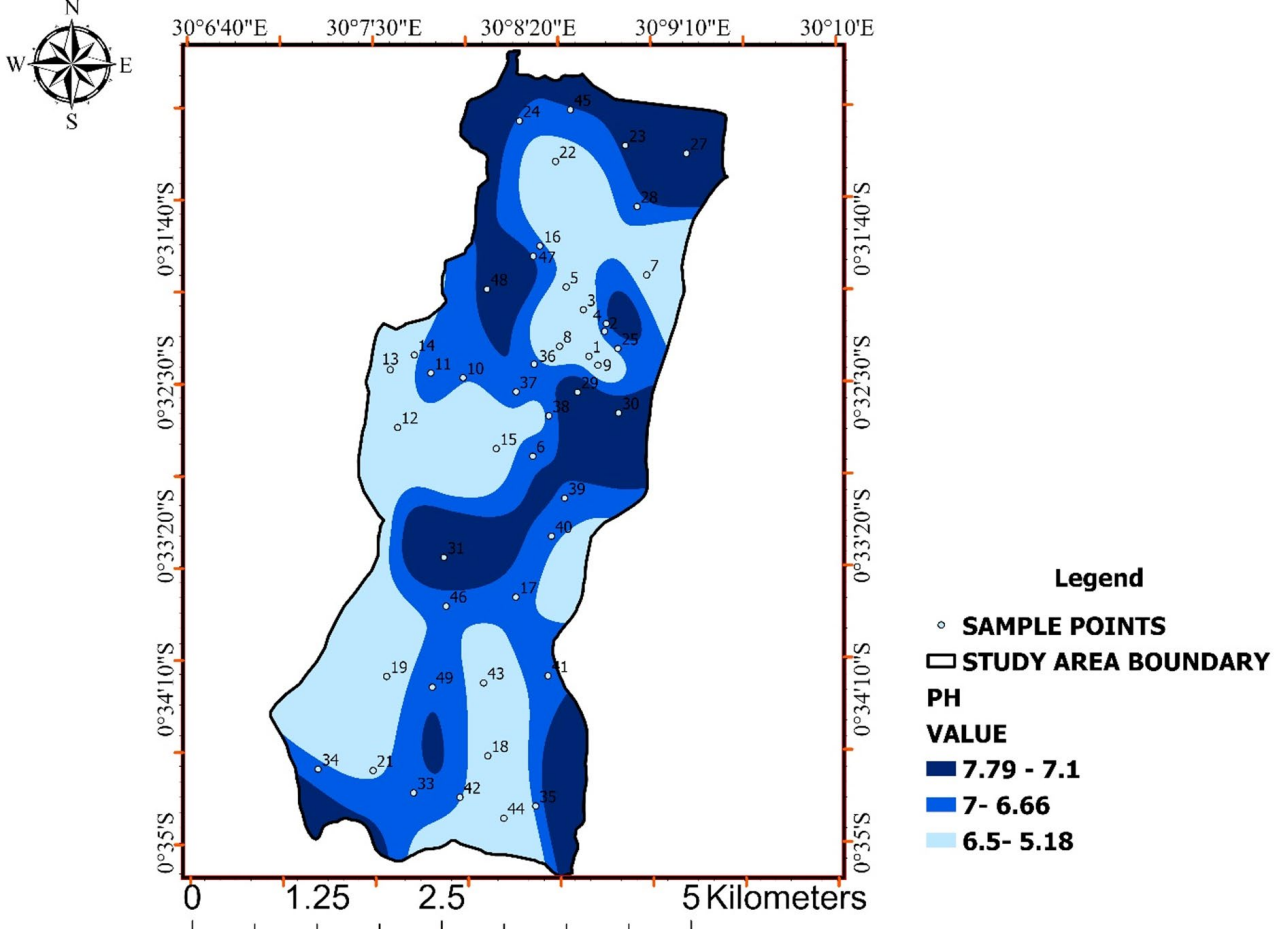
Spatial distribution of pH values across Ishaka Subcounty.

The spatial distribution of pH values (Fig. 2) reveals clear geographic gradients. Neutral to slightly alkaline conditions dominate central and eastern zones, whereas mildly acidic conditions are concentrated in western and southern areas. The absence of abrupt spatial discontinuities suggests that pH variability is governed by gradual geochemical controls, such as soil composition, buffering capacity, and subsurface organic matter decomposition, rather than localized point-source contamination.

Although most samples remain within acceptable limits, zones with acidic pH may increase water corrosivity, potentially enhancing metal leaching from pipes or storage systems. The combined interpretation of Fig. 2 demonstrates the value of spatially resolved analysis for identifying chemically vulnerable zones that may be overlooked by aggregate compliance statistics.

**Table 1:** pH values with WHO and UNBS standards.

Who and Unbs Standard	Result of pH Range
6.5–8.5	5.18–6.5
	6.6 7.0
	7.1–7.79

The map shows interpolated pH values classified according to WHO and UNBS drinking-water standards, highlighting zones with neutral to slightly alkaline conditions and areas exhibiting mildly acidic water chemistry.

### Electrical Conductivity (EC)

Electrical conductivity values ranged from 170 to 512 µS/cm, with a mean of approximately 318 ± 96 µS/cm, remaining well below the WHO guideline value of 1500 µS/cm. These values indicate low to moderate ionic content and generally favourable hadrochemical conditions.

**Fig. 3:**
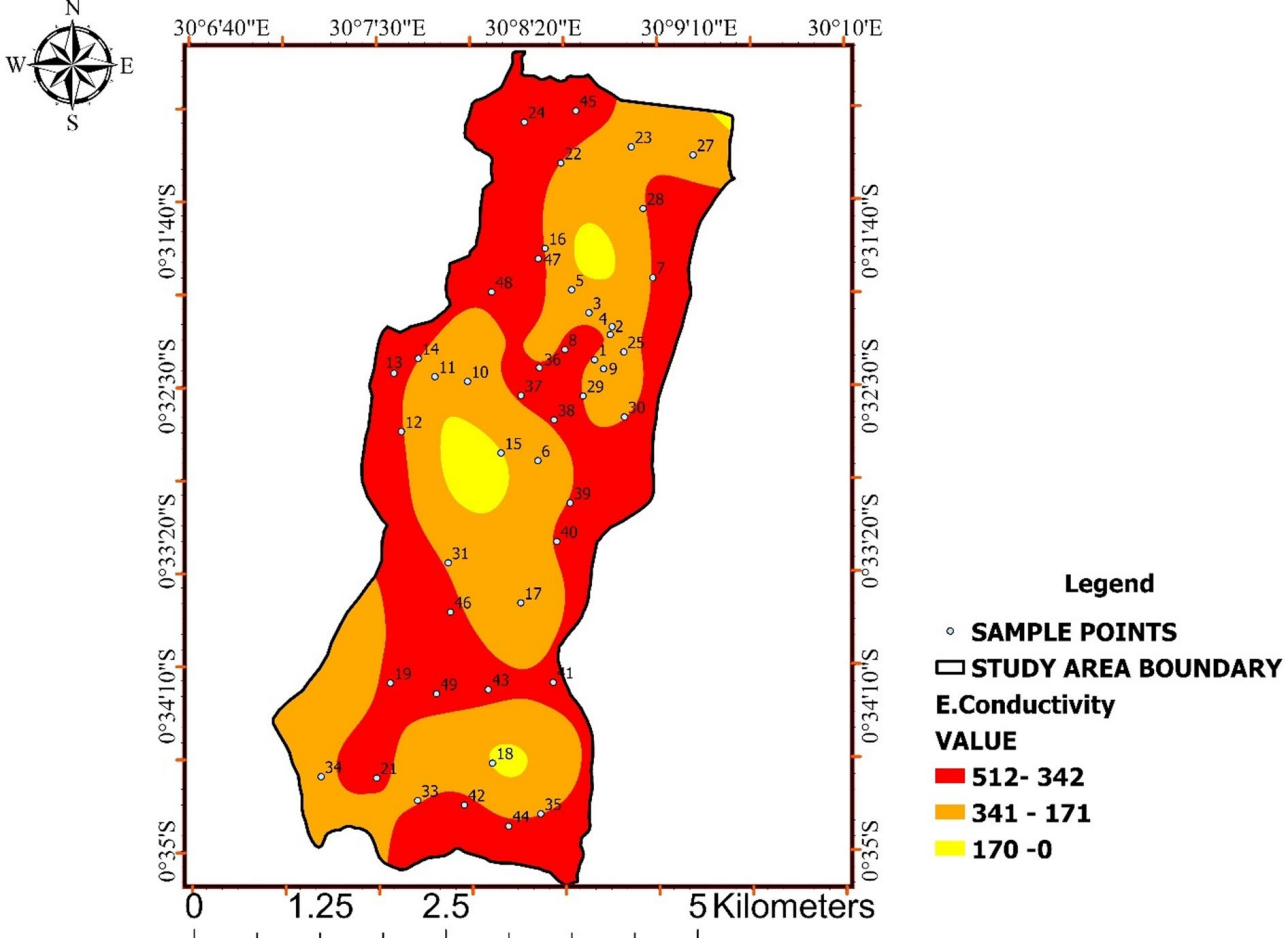
Spatial distribution of electrical conductivity (EC) across Ishaka Subcounty.

As shown in Fig. 3, EC exhibits smooth spatial gradients rather than abrupt changes, suggesting that conductivity variability is primarily controlled by mineral dissolution and groundwater–soil interactions. Areas with relatively elevated EC values likely reflect longer water–rock contact times, whereas lower values indicate limited geochemical interaction.

The absence of extreme EC values suggests minimal influence from salinization or industrial contamination. Together, Table 2; Fig. 3 confirm that EC variability in Ishaka Subcounty reflects natural hydrogeochemical processes rather than anthropogenic stress.

**Table 2:** Electrical conductivity (EC) statistics.

Who and unbs standard	Result of Ec Range (µS/CM)
< 1500	0–170
	171–341
	342–512

Interpolated EC values illustrate spatial variability in ionic content, with all observed concentrations remaining within WHO guideline limits and reflecting natural hydrogeochemical controls.

### Water Temperature

**Fig. 4:**
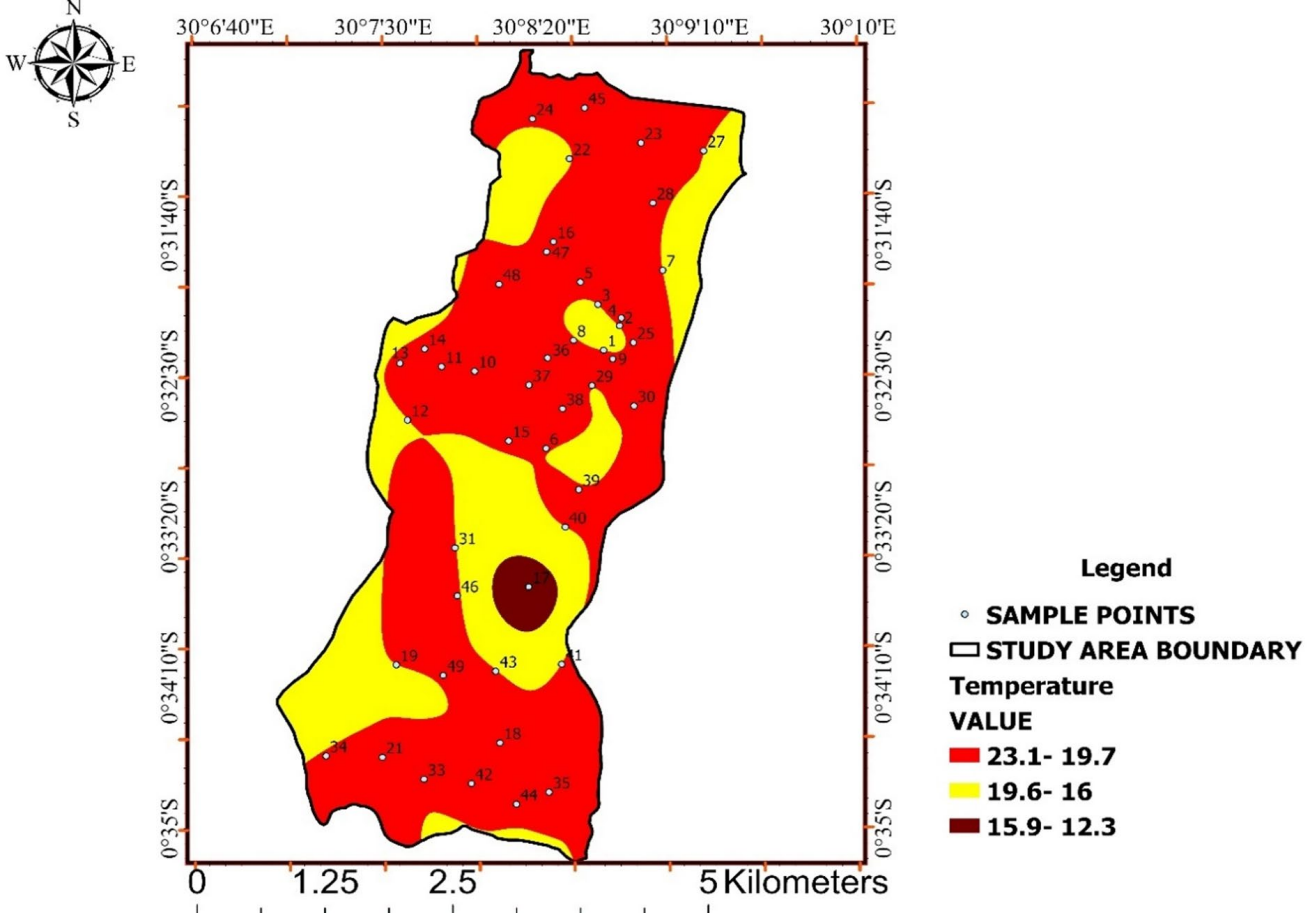
Spatial distribution of water temperature across Ishaka Subcounty.

Spatial patterns in water temperature are evident across Ishaka Subcounty, with peripheral zones generally exhibiting higher values than central areas (Fig. 4). These gradients point to environmental controls such as exposure, shading, and subsurface depth. Measured temperatures ranged from 12.3 °C to 23.1 °C, remaining below the WHO advisory threshold of 25 °C, indicating compliance with drinking-water guidelines as shown in Table 3. Although variation exists, no extreme thermal conditions were observed.

While temperature does not represent a direct chemical hazard, relatively elevated values may enhance microbial survival and regrowth. Thus, temperature acts as a modifying factor influencing biological water quality, a relationship clarified through the combined interpretation of Fig. 4.

**Table 3:** Water temperature distribution relative to WHO guidance.

Who and unbs standard	Result of temp range (°c)
< 25 °C (advisory)	12.3–15.9 °C
	16.0–19.6 °C
	19.7–23.1 °C

The map presents spatial variations in water temperature, showing gradual gradients influenced by environmental exposure and hydrogeological conditions, with all values below the WHO advisory threshold.

### Total Dissolved Solids (TDS)

All measured TDS concentrations were well below the WHO and UNBS guideline value of 1200 mg/L, indicating excellent overall mineral quality. Observed values ranged from 2 to 252 mg/L, reflecting low mineralization across the study area as shown in Table 4.

**Fig. 5:**
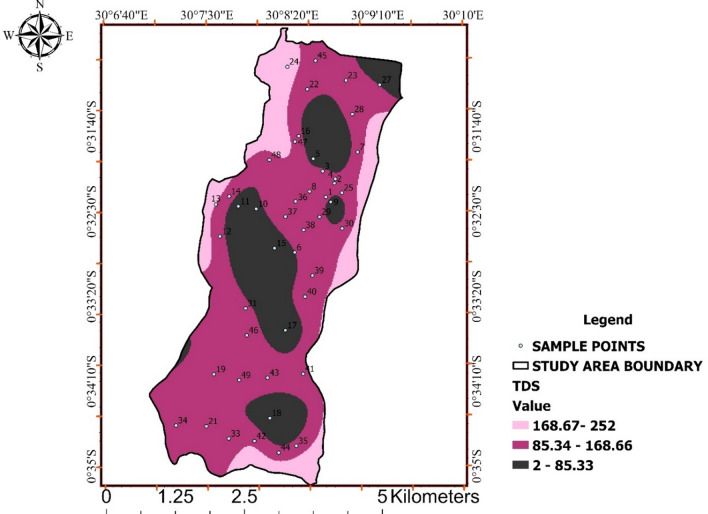
Spatial distribution of total dissolved solids (TDS) across Ishaka Subcounty.

Figure 5 illustrates gradual spatial increases in TDS, consistent with natural mineral dissolution and evaporation processes rather than anthropogenic inputs. The spatial continuity observed suggests diffuse geochemical controls rather than localized contamination. Low TDS levels across large portions of the study area imply limited buffering capacity, which aligns with the occurrence of mildly acidic pH in some zones. The joint interpretation of Fig. 5 provides important hydrogeochemical context for understanding water quality dynamics.

**Table 4:** Result of TDS range (mg/l).

Who and unbs standard	Result of tds range mg/l.
< 1200 mg/L	2–85.33
	85.34–168.66
	168.67–252

Interpolated TDS concentrations indicate low to moderate mineralization across the study area, remaining well within WHO and UNBS drinking-water standards.

### Total Suspended Solids (TSS)

**Fig. 6:**
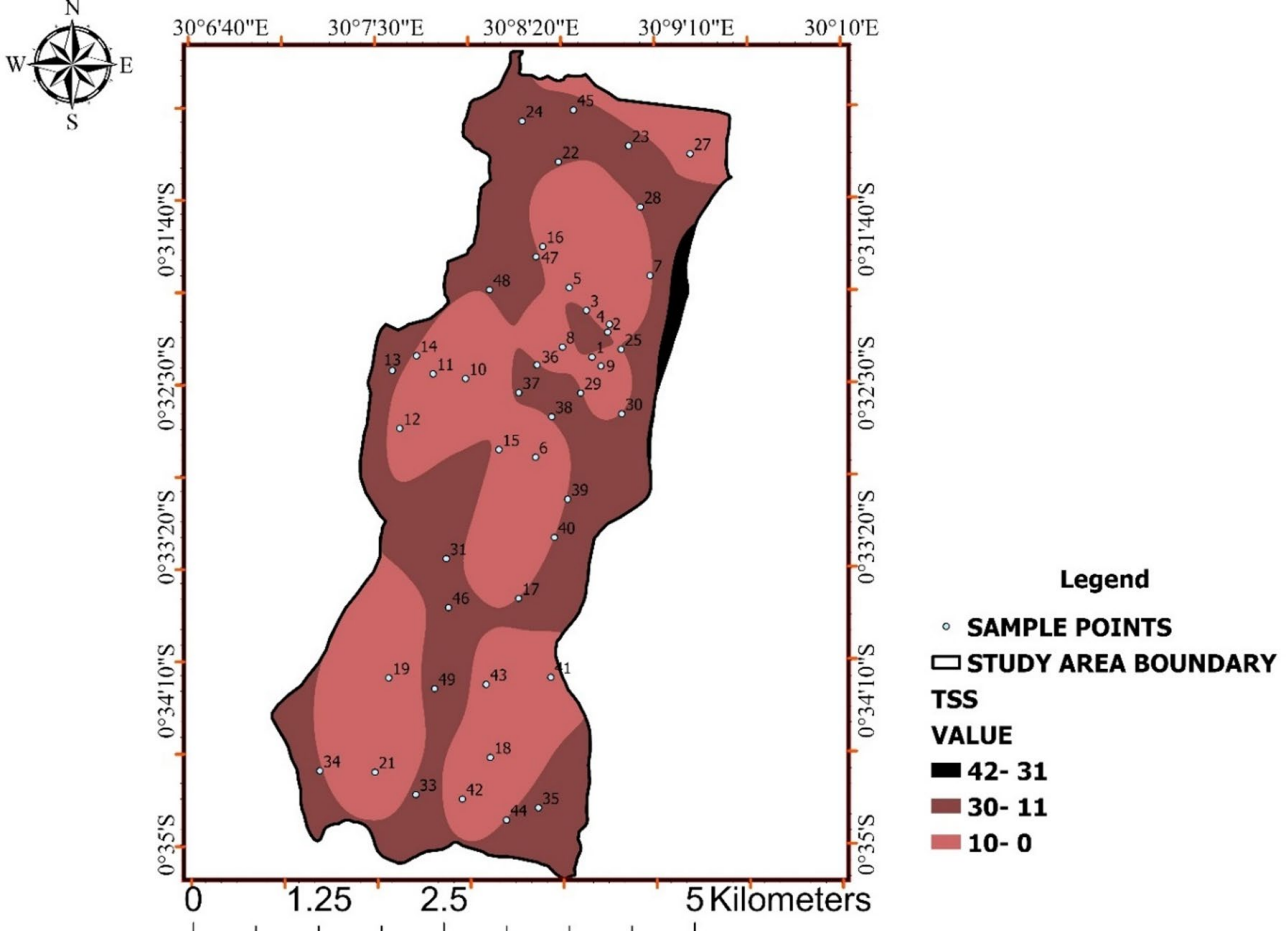
Spatial distribution of total suspended solids (TSS) across Ishaka Subcounty.

Localized zones of elevated suspended solids were identified across Ishaka Subcounty (Fig. 6), contrasting with generally low background concentrations elsewhere.

TSS values ranged from 0 to 42 mg/L, with most samples remaining within the WHO and UNBS acceptable limit of ≤ 30 mg/L, while a subset exceeded this threshold (Table 5). These exceedances indicate localized physical degradation of water quality.

Elevated suspended solids may reduce aesthetic quality and facilitate microbial attachment, indirectly increasing health risks even where chemical parameters remain compliant. Figure 6 together highlight areas where physical water quality constraints may compromise treatment efficiency.

**Table 5:** Total suspended solids (TSS) Values.

Who/unbs limit (mg/l)	TSS (mg/L)
≤ 30	0–10
	11–30
	31–42

The map highlights zones with elevated suspended solids, indicating localized physical water quality degradation potentially associated with runoff and sediment resuspension.

### Nitrate

All nitrate concentrations complied with the WHO drinking-water guideline of 50 mg/L, with measured values ranging from 0.1 to 20 mg/L (Table 6). This indicates the absence of acute nitrate contamination across the study area.

**Fig. 7:**
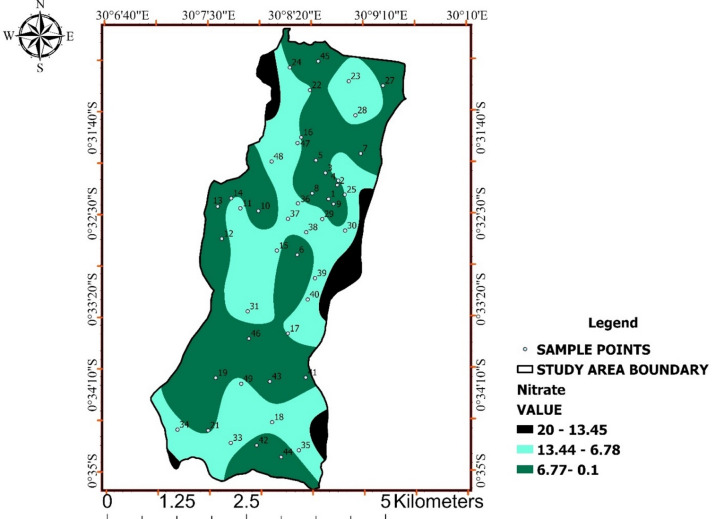
Spatial distribution of nitrate concentrations across Ishaka Subcounty.

Nevertheless, Fig. 7 reveals spatial clustering of relatively elevated nitrate concentrations compared to background levels. These patterns suggest diffuse nutrient inputs rather than point-source contamination.

Although current nitrate levels do not pose immediate health risks, their spatial variability highlights nitrate as an early-warning indicator of emerging nutrient loading. The combined evidence from Fig. 7 supports the need for continued monitoring.

**Table 6:** Nitrate concentration relative to WHO guidelines.

WHO guideline (mg/L)	Nitrate (mg/L)
50	0.1–6.77
	6.78–13.44
	13.45–20.45

Interpolated nitrate levels show spatial variability with generally low to moderate concentrations, remaining below WHO guideline values but indicating potential diffuse nutrient inputs.

### Dissolved Oxygen (DO)

Dissolved oxygen concentrations exhibited substantial spatial variability, ranging from 1.82 to 7.14 mg/L (Table 7). Values below 5 mg/L indicate oxygen stress, while higher values reflect well-oxygenated conditions.

**Fig. 8:**
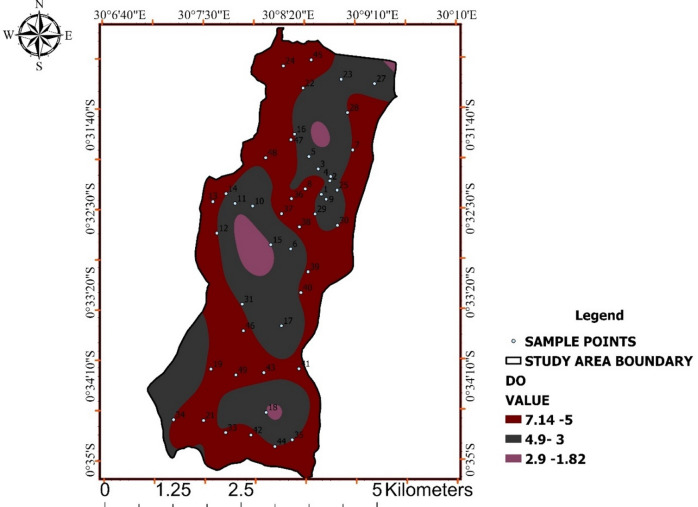
Spatial distribution of dissolved oxygen (DO) across Ishaka Subcounty.

Figure 8 shows clustering of low-DO zones, suggesting reduced water circulation and enhanced organic matter degradation. Such conditions often coincide with increased microbial activity and declining biological water quality.

DO therefore serves as a supporting indicator of ecological and microbiological stress rather than a standalone chemical metric. The integration of Fig. 8 strengthens interpretation of biological vulnerability across the study area.

**Table 7:** Dissolved oxygen (DO) concentration and quality interpretation.

Parameter	Minimum (mg/L)	Maximum (mg/L)	Mean ± SD (mg/L)	Threshold	Interpretation
DO	1.82	7.14	4.9 ± 1.6	≥ 5	Mixed conditions

The map illustrates spatial differences in dissolved oxygen levels, identifying zones of adequate oxygenation and areas exhibiting oxygen stress that may reflect organic matter decomposition and reduced circulation.

### Faecal Coliforms

Microbiological contamination emerged as the most critical water-quality concern in the study. Faecal coliform counts ranged from 0 to > 1,000 CFU/100 mL, with a substantial proportion of samples exceeding WHO and UNBS standards (Table 8).

**Fig. 9:**
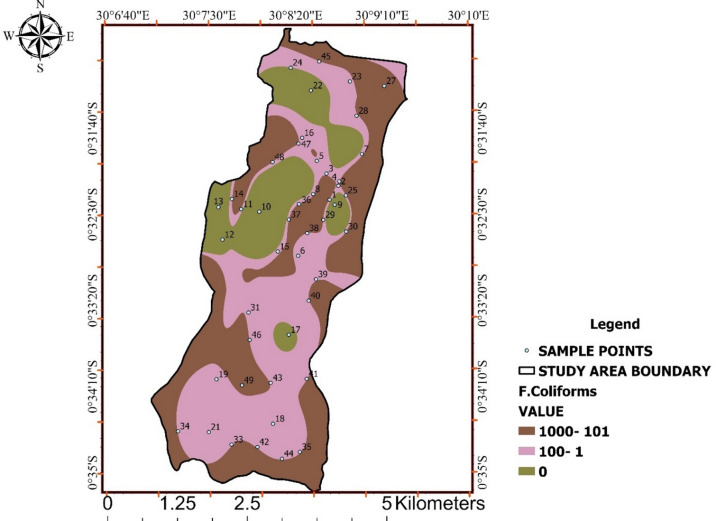
Spatial distribution of faecal coliform concentrations across Ishaka Subcounty.

Spatial analysis (Fig. 9) reveals distinct contamination hotspots rather than uniform distribution, indicating localized faecal inputs and recent contamination events.

Unlike physicochemical parameters, faecal coliforms display sharp spatial contrasts and represent direct public-health risk. The combined interpretation of Fig. 9 underscores the inadequacy of relying solely on chemical compliance to assess drinking-water safety.

**Table 8:** Faecal coliform counts with WHO/UNBS.

Parameter	Range (CFU/100 mL)	WHO/UNBS standard
Faecal coliforms	0 – >1000	0

Spatial patterns reveal clustered faecal contamination hotspots, indicating localized sanitary risks and non-compliance with WHO and UNBS microbiological standards.

### Escherichia coli (E. coli)

E. coli concentrations ranged from 0 to 50 CFU/100 mL, with multiple samples exceeding the zero-tolerance standard recommended by WHO and UNBS (Table 9). These results confirm direct faecal contamination.

**Fig. 10:**
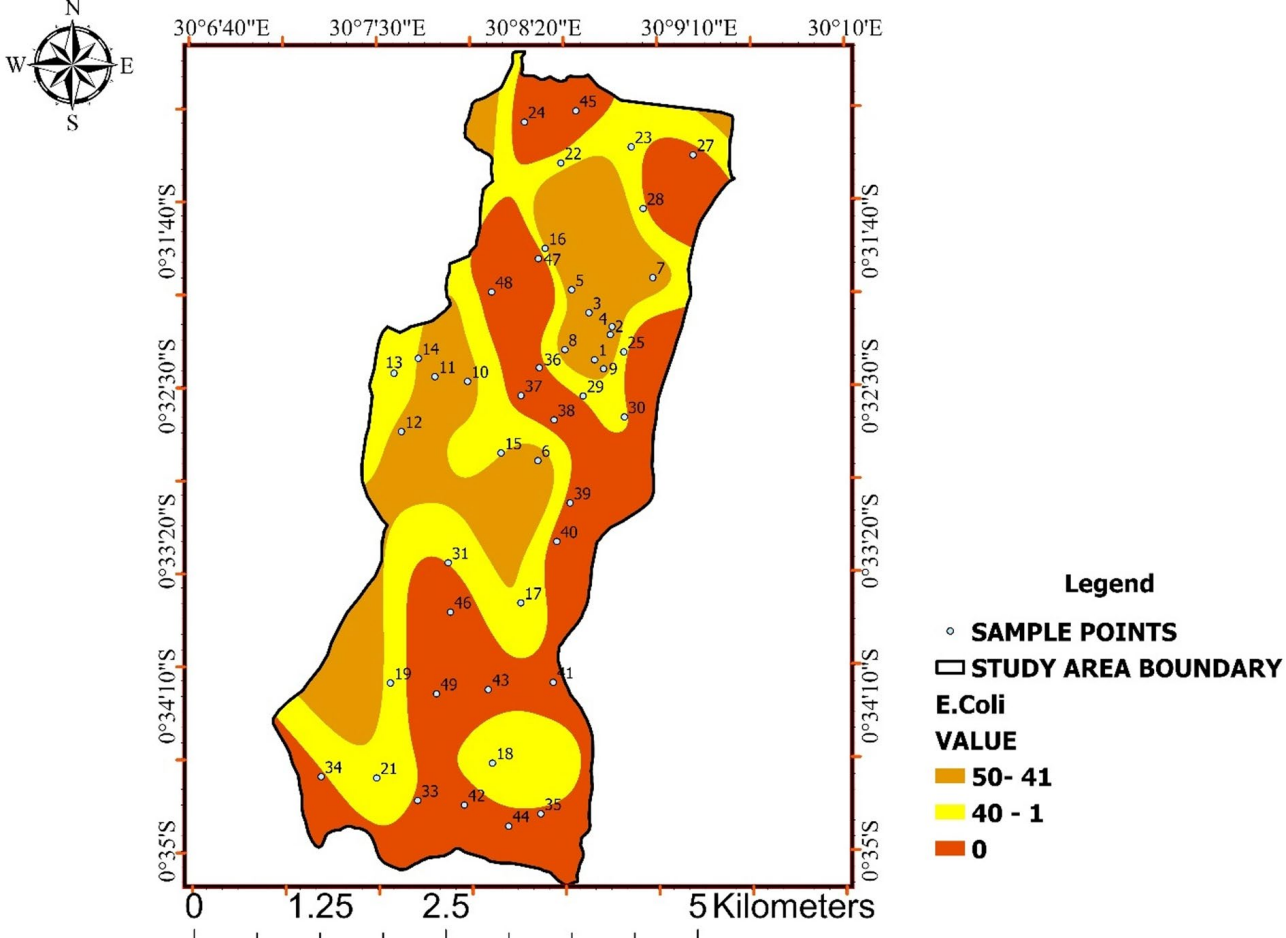
Spatial distribution of *Escherichia coli* (E. coli) concentrations across Ishaka Subcounty.

Figure 10 shows spatial overlap between elevated E. coli levels and broader microbial contamination patterns, indicating systematic rather than random contamination processes.

Even low E. coli counts signify unsafe drinking-water conditions. The combined interpretation of Fig. 10 identifies microbial contamination as the dominant risk factor in Ishaka Subcounty, outweighing physicochemical concerns.

**Table 9:** Escherichia coli (E. coli) occurrence and health risk.

Parameter	Range (CFU/100 mL)	WHO/UNBS standard
E. coli	0–50	0

The map shows areas with detectable *E. coli* contamination, highlighting zones of elevated public health risk associated with direct faecal pollution.

### Descriptive Statistical Summary of Water Quality Parameters

Descriptive statistics were computed to summarize the central tendency and variability of the analysed physicochemical and microbiological parameters across all sampling locations. The results are presented in Table 10.

**Table 10:** Descriptive statistics of water quality parameters in Ishaka Subcounty.

Parameter	Minimum	Maximum	Mean	Standard Deviation
pH	5.18	7.79	6.72	0.61
EC (µS/cm)	170	512	318	96
Temperature (°C)	12.3	23.1	19.4	2.8
TDS (mg/L)	2	252	114	71
TSS (mg/L)	0	42	18	11
Nitrate (mg/L)	0.1	20.0	7.9	5.6
DO (mg/L)	1.82	7.14	4.96	1.31
Faecal coliforms (CFU/100 mL)	0	> 1000	214	356
E. coli (CFU/100 mL)	0	50	9.8	14.2

#### Interpretation of Descriptive Statistics

The mean pH (6.72) fell within WHO and UNBS acceptable limits, indicating generally neutral water conditions, although the relatively high standard deviation reflects localized acidic zones. Electrical conductivity showed a moderate mean value (318 µS/cm) with limited dispersion, supporting the interpretation that dissolved mineral content was low and spatially consistent.

Mean TDS values (114 mg/L) confirmed low mineralization, while the higher standard deviation suggests spatial heterogeneity driven by geological variability. In contrast, microbiological parameters exhibited the highest variability, with faecal coliforms and *E. coli* showing large standard deviations relative to their means. This pattern indicates highly localized contamination hotspots, rather than uniform microbial degradation.

The mean dissolved oxygen concentration (4.96 mg/L) was close to the recommended threshold, but the minimum value (1.82 mg/L) highlights zones experiencing oxygen stress. Overall, descriptive statistics corroborate spatial analysis results, confirming that chemical quality is largely compliant, whereas microbial contamination represents the primary health risk.

### Compliance Summary with WHO and UNBS Standards

The Compliance summary of water quality parameters with WHO and UNBS using total number of samples 49 as shown in Table 11.

**Table 11:** Summary of compliance of key water quality parameters with WHO and UNBS drinking-water standards (*n* = 49).

Parameter	WHO/UNBS Guideline	Compliant (*n*, %)	Non-compliant (*n*, %)	Interpretation
pH	6.5–8.5	36 (73%)	13 (27%)	Majority within acceptable range; localized acidic conditions present
Electrical Conductivity (EC)	< 1500 µS/cm	49 (100%)	0 (0%)	Fully compliant; low ionic content
Temperature	< 25 °C (advisory)	49 (100%)	0 (0%)	Within advisory range; may influence microbial regrowth
Total Dissolved Solids (TDS)	< 1200 mg/L	49 (100%)	0 (0%)	Excellent mineral quality
Total Suspended Solids (TSS)	≤ 30 mg/L	34 (69%)	15 (31%)	Localized exceedances indicating physical water quality degradation
Nitrate (NO₃⁻)	≤ 50 mg/L	49 (100%)	0 (0%)	No acute nitrate contamination
Dissolved Oxygen (DO)	≥ 5 mg/L	22 (45%)	27 (55%)	Mixed oxygenation; zones of oxygen stress present
Faecal coliforms	0 CFU/100 mL	11 (22%)	38 (78%)	Widespread faecal contamination
*Escherichia coli*	0 CFU/100 mL	19 (39%)	30 (61%)	Direct faecal contamination detected

### Correlation Analysis

Pearson correlation analysis was conducted to examine relationships among selected parameters. A strong positive correlation was observed between EC and TDS (*r* > 0.8), confirming that ionic strength was controlled by dissolved solids. A moderate positive correlation between TSS and faecal coliforms suggests that suspended particles may facilitate microbial transport and persistence.

No statistically meaningful correlation was observed between pH and microbial indicators, indicating that faecal contamination was not driven by acidity. These statistical relationships strengthen the interpretation of contamination dynamics and support the spatial patterns observed.

## Conclusion

This study assessed the physicochemical and microbiological quality of drinking water in Ishaka Subcounty, Bushenyi District, Uganda, using field measurements, laboratory analysis, and GIS-based spatial mapping. The findings indicate substantial spatial variability in water quality across the study area, reflecting differences in source protection, treatment reliability, and surrounding sanitation conditions.

Overall, key physicochemical parameters largely complied with national and World Health Organization guideline values. However, localized deviations such as mildly acidic pH were identified, suggesting potential operational or geochemical influences that may affect drinking-water suitability in specific zones. In contrast, microbiological indicators showed widespread contamination, highlighting a significant public health concern even where chemical quality appeared acceptable. The observed association between suspended solids and microbial indicators further suggests that surface infiltration and physical disturbance may contribute to contamination pathways.

GIS-based spatial analysis proved valuable for identifying contamination hotspots and visualizing spatial patterns of water quality degradation. Even without advanced validation metrics, the interpolation surfaces provided useful exploratory insights into high-risk zones that may not be apparent from point-based assessments alone. Although the study was cross-sectional and represents a snapshot of conditions during the sampling period, the integrated approach provides a robust baseline for improving monitoring, strengthening water safety interventions, and supporting evidence-based public health planning. Strengthened routine monitoring, improved treatment reliability, enhanced source protection, and community hygiene promotion remain critical for reducing health risks and improving the sustainability of drinking-water services in the study area.

## Recommendations

Focusing on the health risks associated with unsafe water, open defecation, and improper waste disposal would contribute to reducing contamination at both source and household levels. Engagement of local leaders and health workers is critical to ensure sustained behavioural change.

### Short-Term Actions

- Distribute chlorine tablets and promote boiling/SODIS in contamination hotspots.
- Conduct rapid sanitary inspections at all high-risk water points.
- Teach safe water handling: clean containers, covered storage, handwashing.
- Add limestone to treat acidic water in western and southern zones.
- Start quarterly E. coli testing for all improved water sources.

### Long-Term Actions

- Upgrade vulnerable sources: box springs, line wells, install fencing.
- Enforce 30-metre sanitation rule and require lined latrines near water points.
- Improve piped water reliability through storage and pressure management.
- Train local staff in GIS and maintain georeferenced water quality database.
- Make E. coli testing mandatory in national monitoring guidelines.
- Establish year-round monitoring to capture seasonal variation.

## Limitations

This study has several limitations that should be considered when interpreting the findings. First, the study adopted a cross-sectional design, providing a snapshot of water quality conditions at a single point in time. As a result, seasonal and temporal variations, particularly between wet and dry seasons, were not captured and may influence both physicochemical and microbiological parameters.

Second, although the sampling strategy aimed to maximize spatial coverage and public health relevance, the number of sampling sites (*n* = 49) may not fully represent all localized contamination dynamics within the subcounty. In particular, micro-scale sanitary risks associated with specific households or facilities may not be fully reflected.

Third, spatial interpolation using the Thin Plate Spline (TPS) method was applied for exploratory mapping purposes without formal cross-validation metrics such as RMSE. Consequently, the generated spatial surfaces should be interpreted as indicative patterns rather than precise predictive estimates.

Finally, the analytical scope focused on core physicochemical and microbiological indicators. Potentially important contaminants such as heavy metals, pesticides, pharmaceuticals, and microplastics were not assessed, and sanitary inspection scores were not quantitatively evaluated. These limitations highlight areas where future research can strengthen understanding of water quality risks in the study area.

## Directions for Future Research

This study adopted a cross-sectional design and therefore provides a snapshot of water quality conditions in Ishaka Subcounty. Future research should incorporate longitudinal monitoring to capture seasonal and inter-annual variations in both physicochemical and microbiological parameters, particularly across wet and dry seasons.

Furthermore, while this study focused on core indicators such as pH, EC, TDS, DO, nitrates, and coliform bacteria, future investigations should expand the analytical scope to include heavy metals (e.g., lead, arsenic, mercury) and emerging contaminants such as pesticides, pharmaceutical residues, and microplastics. Such expanded assessments would enable a more comprehensive evaluation of water safety and associated public health risks.

## Data Availability

All data generated or analyzed during this study are included in this article.
